# PMGen: from peptide-MHC structure prediction to peptide generation

**DOI:** 10.1093/bioinformatics/btag381

**Published:** 2026-06-15

**Authors:** Amir H Asgary, Amirreza Aleyasin, Jonas A Mehl, Salman S Fallah, Hasmig Aintablian, Burkhard Ludewig, Michele Mishto, Juliane Liepe, Johannes Söding

**Affiliations:** Quantitative and Computational Biology Group, Max Planck Institute for Multidisciplinary Sciences, Göttingen 37077, Germany; Quantitative and Computational Biology Group, Max Planck Institute for Multidisciplinary Sciences, Göttingen 37077, Germany; Quantitative and Computational Biology Group, Max Planck Institute for Multidisciplinary Sciences, Göttingen 37077, Germany; Quantitative and Computational Biology Group, Max Planck Institute for Multidisciplinary Sciences, Göttingen 37077, Germany; Quantitative and Computational Biology Group, Max Planck Institute for Multidisciplinary Sciences, Göttingen 37077, Germany; Institute of Immunobiology, Kantonsspital St. Gallen, St. Gallen 9007, Switzerland; Centre for Inflammation Biology and Cancer Immunology (CIBCI) & Peter Gorer Department of Immunobiology, King’s College London, London SE1 1UL, United Kingdom; Research Group of Molecular Immunology The Francis Crick Institute, London NW1 1AT, United Kingdom; Max Planck Institute for Multidisciplinary Sciences, Göttingen 37077, Germany; Quantitative and Computational Biology Group, Max Planck Institute for Multidisciplinary Sciences, Göttingen 37077, Germany

## Abstract

**Motivation:**

Accurate structural modeling of peptide–major histocompatibility complex (pMHC) complexes is essential for structure-driven immunotherapy design, yet current prediction tools suffer from narrow class coverage, restricted peptide lengths, insufficient accuracy, and a lack of built-in structure-aware peptide sampling. Consequently, most mimotope and altered peptide ligand designs rely solely on sequence substitution, leaving spatial and biophysical insights from pMHC structures largely unexploited.

**Results:**

We introduce peptide–MHC generator (PMGen), an integrated framework for structure prediction and structure-guided design of variable-length peptides across MHC Class I and II. PMGen enforces anchor constraints within AlphaFold2 through two complementary strategies, initial guess and template engineering, achieving state-of-the-art structural fidelity without model fine-tuning. On a comprehensive benchmark, PMGen outperforms all existing methods, yielding median peptide-core Cα RMSDs of 0.62 Å for MHC-I and 0.33 Å for MHC-II. We show that PMGen can recover incorrectly predicted anchor positions and that AlphaFold pLDDT scores enable sequence-independent binding-core identification. Applied to a published neoantigen/wild-type pair, PMGen accurately captures mutation-induced conformational changes. Beyond structure prediction, we show that ProteinMPNN sampling on PMGen-predicted backbones yields higher affinity peptides while preserving the parental 3D conformation. Using PMGen to generate 63 817 high-confidence pMHC structures as training data, we further improve ProteinMPNN’s peptide sequence recovery from 0.14 to 0.64 on a test set of 85 unseen MHC-I alleles, highlighting the value of accurate predicted structures for downstream machine learning tasks.

**Availability and implementation:**

PMGen is freely available at https://github.com/soedinglab/PMGen, with an interactive Colab notebook at https://colab.research.google.com/github/soedinglab/PMGen/blob/master/colab.ipynb.

## 1 Introduction

Major histocompatibility complexes (MHCs) are central to adaptive immunity. They present intracellular (MHC-I) or extracellular (MHC-II) peptides to T cells to trigger specific immune responses (Barbosa *et al.* 2021). Antigenic peptides are vital for orchestrating immune activity, and their generation and presentation pathways are tightly regulated to ensure specificity and prevent autoimmunity. This regulation involves the generation of peptide antigens, transport and selective peptide binding to MHC molecules, activation of T cell receptors (TCRs), and modulation by regulatory T cells. When this balance is disrupted, such as when self-peptides are misidentified as foreign, autoimmune diseases can result (Rojas *et al.* 2024).

Designing peptides that bind effectively to MHC molecules has direct applications for immunotherapy and vaccine development. Identifying or engineering antigenic peptides with high binding affinity to specific MHC alleles enables the development of personalized treatments for cancer and infectious diseases. Immune checkpoint therapies, for example, have shown remarkable success in certain cancer patients by leveraging MHC-I-restricted neoantigen responses mediated by ${\text{CD}8}^{+}$ T cells (Matsushita *et al.* 2012, Robbins *et al.* 2013, Gubin *et al.* 2014, Snyder *et al.* 2014, Strønen *et al.* 2016, Wieder *et al.* 2018, Dolina *et al.* 2023), and ${\text{CD}4}^{+}$ T cell activation is at the cutting edge of targeted anticancer immunotherapies (Kreiter *et al.* 2015, Ott *et al.* 2017, Alspach *et al.* 2019).

While tumors naturally present mutated neoepitopes, many are ineffective targets due to poor processing or weak MHC binding. In such cases, *in silico* redesign can improve immunogenicity while preserving structural similarity to the native antigen. A notable example is the engineered T210M variant of the melanoma-associated gp100 epitope, designed to enhance binding to human leukocyte antigen (HLA)-A*02:01 (Leonhardt *et al.* 2011). To automate this discovery, reinforcement learning (RL)-based generators such as PepPPO (Chen *et al.* 2023) and RLpMIEC (Deng *et al.* 2024) have been proposed. These approaches, alongside established sequence-based predictors like NetMHCpan (Hoof *et al.* 2009, Nilsson *et al.* 2025) and others (Jin *et al.* 2021, Kalemati *et al.* 2023, Qu *et al.* 2023, Thrift *et al.* 2024), learn from large-scale pMHC binding data. However, their utility in personalized medicine is often constrained by the inherent bias and class imbalance of training datasets; they perform well on common alleles but frequently fail to generalize to rare or novel MHC alleles where data are scarce.

**Figure 1:**
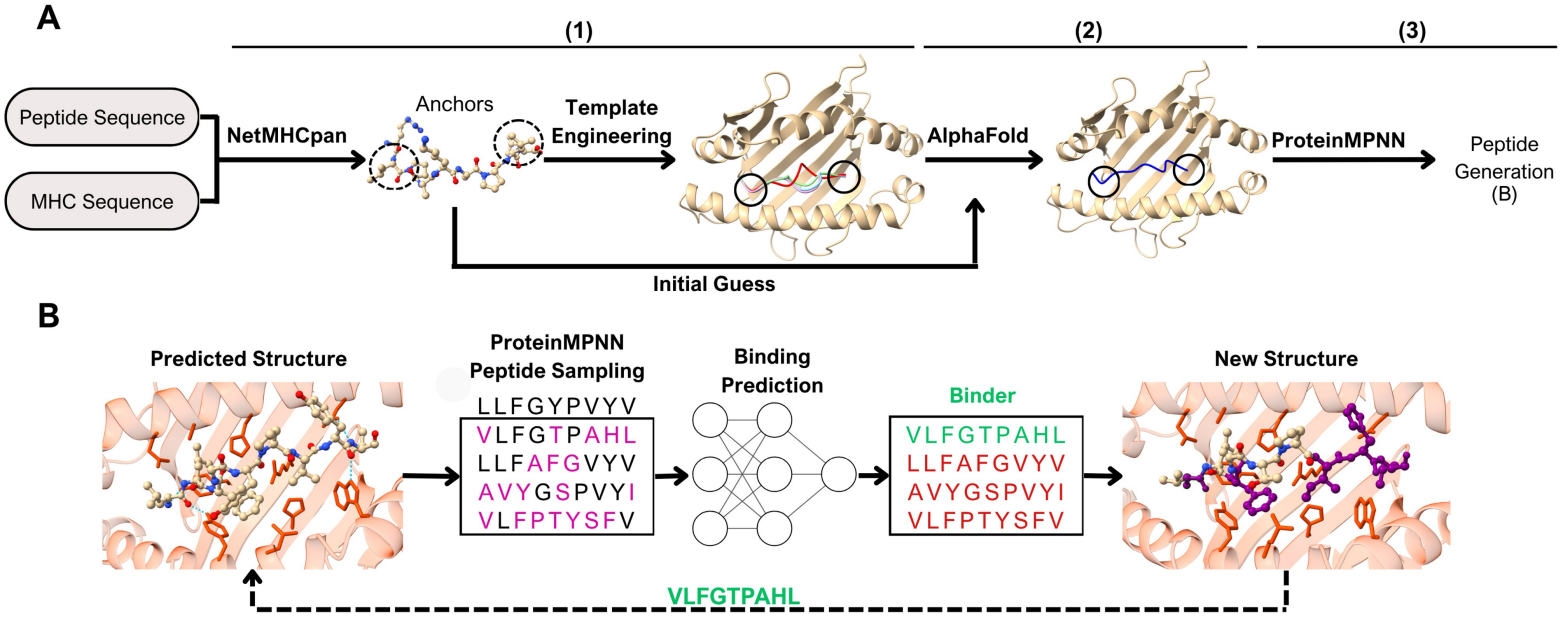
Anchor-guided modeling and structure-based peptide generation in PMGen. (A) For a given peptide–MHC pair, PMGen predicts anchor positions using NetMHCpan. Initial structural coordinates (initial guess) or engineered templates (with peptides from different templates shown in color) are used to input anchor position information to AlphaFold (1). AlphaFold performs anchor-aware structure prediction (2). The resulting backbone serves as input for ProteinMPNN to design alternative peptides (3). (B) Multiple peptide sequences are sampled using ProteinMPNN, and their binding affinities are predicted. The top-scoring peptide is then used for another round of structure prediction. This process can be repeated iteratively. Dashed lines represent the iterative loop, aiming to converge toward a high-affinity binder with an optimized sequence.

Structural modeling offers a pathway to overcome these sequence-based limitations by leveraging the conserved architecture of the MHC binding groove. Both MHC classes share a similar fold: a β-sheet base flanked by two α-helices, with the peptide nestled between them (Wieczorek *et al.* 2017), though they differ in groove geometry and peptide conformation (Fig. 1, available as supplementary data at *Bioinformatics* online). Peptide binding is primarily driven by “anchor residues” that interact tightly with MHC pockets. Classical anchors include P2 and PΩ for MHC-I (Bouvier and Wiley 1994, Zacharias and Springer 2004), and P1, P4, P6, and P9 for MHC-II (Chicz *et al.* 1992), with each allele having a preferred range of peptide lengths (Sarkizova *et al.* 2020). Accurate modeling of these interactions is essential to study the peptide mutation impact on T cell recognition (Xia *et al.* 2023). Furthermore, restricting modeling to canonical anchor positions has been shown to significantly reduce peptide-core RMSD (Marzella *et al.* 2022). Recent work has demonstrated that such structural representations can be effective for sampling peptides under constraints beyond the MHC alone, including the TCR context (Visani *et al.* 2025). Consequently, accurate pMHC structure prediction could provide the consistent training signals needed to build transferable models that generalize across MHC alleles and classes.

Despite this potential, accurate pMHC modeling remains a computational bottleneck. MHC-II modeling is particularly complex due to its dual-chain architecture and variable peptide length (11–25 residues), while MHC-I modeling struggles with predicting the flexible central core of the peptide (Ko *et al.* 2024). Although AlphaFold (Abramson *et al.* 2024) excels at protein folding, it often fails to correctly dock bound peptides (Harmalkar *et al.* 2025). Recent methods like Tfold (Mikhaylov *et al.* 2024), MHC-Fine (Glukhov *et al.* 2024), and PANDORA (Marzella *et al.* 2022) attempt to address this by incorporating anchor information or fine-tuning. However, these tools face limitations such as restricted peptide length coverage, lack of MHC-II support, or high computational costs that hinder large-scale screening.

To address these limitations, we introduce peptide–MHC generator (PMGen; https://github.com/soedinglab/PMGen), a unified pipeline for pMHC modeling and peptide design across both MHC classes. PMGen integrates anchor-guided AlphaFold modeling with structure-aware peptide generation. In benchmark evaluations, PMGen outperformed current state-of-the-art structure predictors. By bridging the gap between sequence-based prediction and structure-based optimization, PMGen provides a versatile platform for immunotherapy design, neoantigen modeling, and structural immunology.

## 2 Materials and methods

### 2.1 pMHC protein data bank data processing

**Figure 2:**
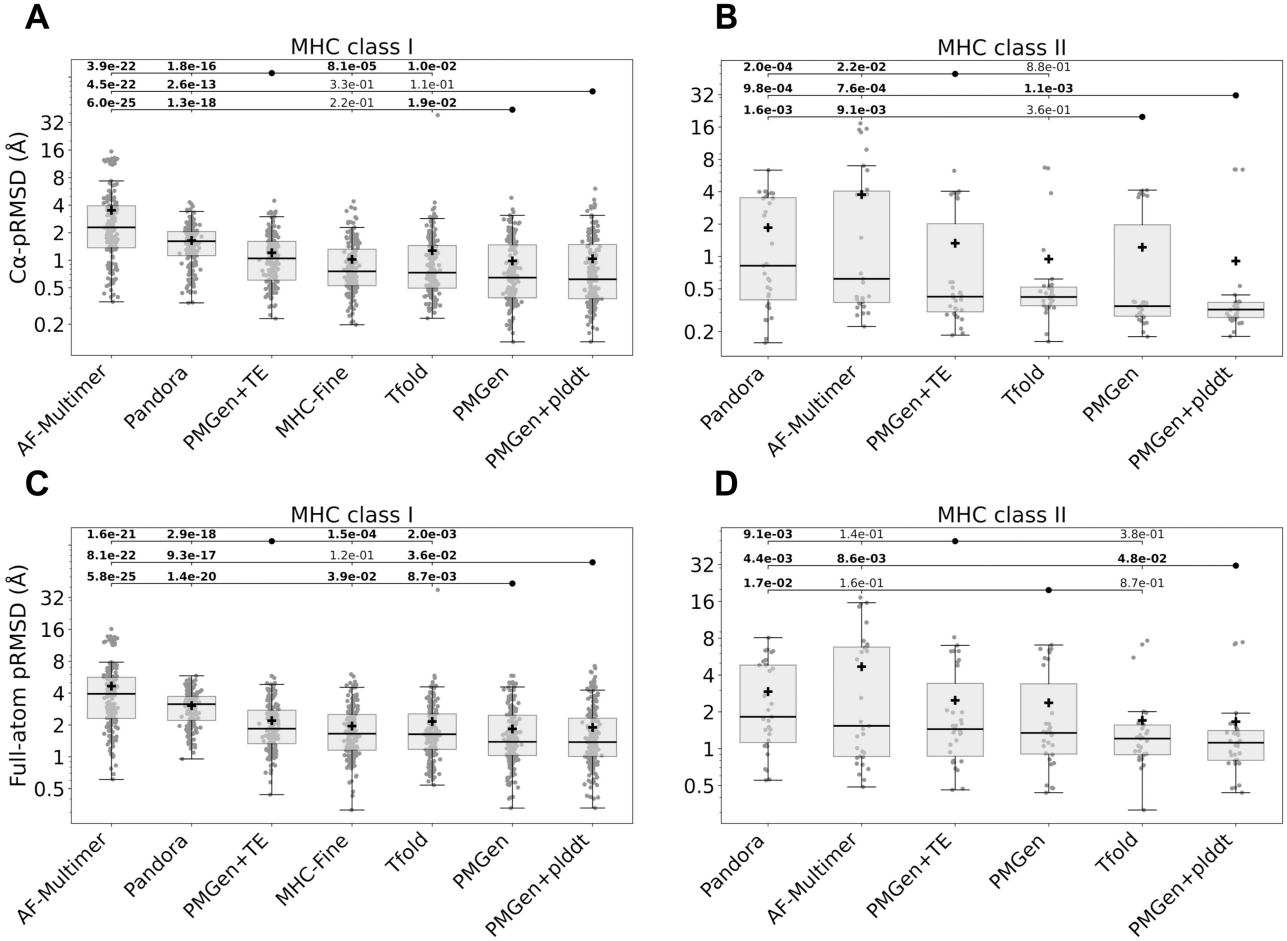
Comparative modeling assessment. Modeling performance measured by RMSD over peptide Cα atoms (A, B) and full atoms (C, D). Panels (A) and (C) display results for MHC-I (*n* = 176), while panels (B) and (D) display results for MHC-II (*n* = 31). Medians and means are indicated by solid lines (−) and crosses (+), respectively. The *P-*value from Wilcoxon signed-rank tests, comparing each method against the PMGen variants, are shown above the plots, with significant values highlighted in bold. PMGen represents the default initial guess mode; PMGen+TE utilizes template engineering; PMGen+pLDDT represents initial guess mode with anchor selection based on pLDDT scores.

We collected protein data bank (PDB) files containing bound-state peptide–MHC structures from the ImMunoGeneTics information system (Lefranc 2011) and RCSB (https://www.rcsb.org/) (Research Collaboratory for Structural Bioinformatics 2025) databases. Structures with a reported resolution worse than 3.5 Å were excluded consistent with prior pMHC benchmarks (Glukhov *et al.* 2024). All PDB files were cleaned and renumbered, and nonamino acid molecules were removed using the Biopython PDB module (Cock *et al.* 2009). This processing yielded 1144 structures. We subsequently retained only those deposited after the AlphaFold 2.2 training cutoff date of 30 April 2018, leaving 352 structures. We then applied a pipeline incorporating sequence and structural alignment to extract a single pMHC structure from each deposited PDB complex. For structural alignment, each chain of the collected PDB structures was aligned against the corresponding chain of selected reference structures: 4H25 for MHC-II and 4U6Y for MHC-I. Immunoglobulin-like domains (β2 and α2 for MHC-II; α3 for MHC-I) were manually removed from the references, retaining only the peptide-binding domains. Structural alignment was performed exclusively on these binding domains using TMalign (Zhang and Skolnick 2005), after which all nonaligned MHC regions were discarded. In cases where PDB files contained additional chains (e.g. TCRs), only the peptide and MHC chains were retained. The peptide chain was identified as the chain with length ≤25 and >7 amino acids and having the smallest Euclidean distance to the center of mass of the MHC binding domain. After manually verifying the chain assignments, we performed an all-versus-all sequence alignment to identify duplicates—structures sharing identical peptides and MHCs from the exact same allele—and retained only one representative from each set. Overall, cases involving overly long or short peptides, broken chains, nonstandard amino acids, structures wrongly assigned as MHC (MHC-like structures), and redundant entries were removed (Table 1, available as supplementary data at *Bioinformatics* online). A final dataset of 32 MHC-II and 187 MHC-I structures released after the training cutoff date was used for our benchmarking and analysis, while those released before this date were assigned to a discovery set used to identify the best AlphaFold model parameters (Fig. 2, available as supplementary data at *Bioinformatics* online). When subsequently running other benchmarking tools, 12 structures were excluded from the comparative benchmarking (11 MHC-I and 1 MHC-II) due to failures encountered with some of the tools.

### 2.2 Template engineering

PMGen takes peptide and MHC sequences as input to predict the pMHC structure and binding affinity, with an option for peptide generation. The workflow begins by identifying potential peptide anchor positions, which are either predicted by NetMHCpan or specified by the user. For long peptides with multiple predicted anchors, users can retain the top *k* anchors ranked by NetMHCpan’s %EL and binding affinity (see Supplementary Methods, available as supplementary data at *Bioinformatics* online). In this study, we used only the top-ranked anchor for benchmarking.

To identify the MHC allele, PMGen aligns the query MHC sequence against a local repository of MHC sequences with known structures, selecting the closest match by sequence similarity. Alternatively, the allele can be user-specified. If the identified allele is present in NetMHCpan’s supported list, it is used directly for anchor prediction. Otherwise, PMGen selects the most similar supported allele based on alignment score solely for anchor prediction, preserving the original query sequence for modeling.

The query sequences and defined anchor positions are then processed by a modified PANDORA pipeline (Marzella *et al.* 2022) (see Supplementary Methods, available as supplementary data at *Bioinformatics* online). PANDORA performs a BLAST search against the local pMHC database to select the top homologous structure, identifies the MHC anchor-binding pockets, and uses the coordinates of the peptide’s anchor residues as spatial constraints. Anchor-constrained homology modeling is subsequently performed using MODELLER (Webb and Sali 2016).

We term these constrained structures *engineered templates*. While the peptide geometry within the MHC binding groove (anchors) remains consistent across templates, the core and flanking regions vary. PMGen generates multiple engineered templates per query (default: four), ranking them by MODELLER’s molpdf score, and supplies them as input templates to AlphaFold.

### 2.3 AlphaFold initial guess implementation

To reduce computational overhead and guide peptide docking, PMGen utilizes an “initial guess” (IG) mode inspired by Bennett *et al.* (2023). First, a BLAST search against a local pMHC PDB database identifies the most similar structures. We then create a custom pMHC alignment from these templates. Peptide anchor positions are aligned first as fixed positional reference points, followed by the remaining residues. Next, we extract the 3D coordinates from the highest-scoring template to serve as our starting structure. To hold the anchors in place while allowing the rest of the peptide to remain flexible, we mask only the nonanchor positions. The exact 3D coordinates for all MHC residues and the aligned peptide anchors are retained, while the coordinates for the nonanchor peptide residues are masked. These modified coordinates are fed directly into the AlphaFold Model Runner alongside standard inputs. We modified the source code so that the first-recycle input variable is initialized with these custom coordinates instead of the default zeros. Internally, AlphaFold converts this variable into a distance map. By using our custom initialization, we explicitly encode the spatial distances of the peptide anchors relative to the MHC. Because the nonanchor positions are masked, they remain structurally unconstrained. Since the number of anchors is always 2 for MHC-I and 4 for MHC-II, this approach is robust to sequence length differences between the selected template and the query sequence. This forces AlphaFold to dynamically fold the peptide core from scratch during its initial prediction iteration, right before standard refinement begins.

## 3 Results

### 3.1 PMGen pipeline

PMGen consists of three core components: (i) the anchor feeding module, (ii) structure prediction, and (iii) peptide generation/binder selection (Fig. 1A). The anchor feeding module takes peptide and MHC sequences as input, predicts anchor residues with NetMHCpan (Nilsson *et al.* 2025), and provides these as constraints to AlphaFold2 (Jumper *et al.* 2021). Alternatively, anchors can be specified by the user or selected based on the highest pLDDT score. In the structure prediction module, we implemented two alternative approaches for incorporating anchor information into AlphaFold. First, IG, which initializes AlphaFold2’s structure module with peptide anchor positions derived from templates with aligned anchors. Second, template engineering (TE), which performs anchor-constrained homology modeling using PANDORA, where the generated homology models are supplied to AlphaFold’s template module in place of real protein structures (see Section 2). For peptide generation (Fig. 1B), PMGen employs ProteinMPNN (Dauparas *et al.* 2022) to sample alternative peptides that align with the predicted backbone, either in a single step or iteratively. The sampled peptides can then be selected based on their predicted binding affinity to the MHC.

### 3.2 Peptide–MHC modeling benchmark

To assess PMGen’s modeling performance, we compared it against four state-of-the-art tools: PANDORA (Marzella *et al.* 2022), Tfold (Mikhaylov *et al.* 2024), AlphaFold Multimer (Evans *et al.* 2021), and MHC-Fine (Glukhov *et al.* 2024). We benchmarked PMGen in three modes: (i) PMGen, which denotes the default IG mode with the top NetMHCpan-predicted anchor; (ii) PMGen+TE, which uses TE instead of IG; and (iii) PMGen+pLDDT, which enumerates all valid anchor combinations in IG mode and selects the structure with the highest pLDDT, bypassing NetMHCpan-based anchor selection (see Supplementary Methods, available as supplementary data at *Bioinformatics* online). To exclude any structures used in AlphaFold2 training, we tested only on structures released after the cutoff date of 30 April 2018. Since AlphaFold3 (Abramson *et al.* 2024) and the latest version of AF-Multimer have more recent training cutoffs, we utilized AF-Multimer v2.2 for benchmarking. We used AlphaFold model_2_ptm parameters for PMGen predictions due to its higher average pLDDT (Fig. 2, available as supplementary data at *Bioinformatics* online) on pMHC structures released before the benchmarking cut-off date (see Materials and methods).

PMGen and PMGen+pLDDT outperformed all other methods across both MHC classes, achieving median peptide root-mean-square deviation (pRMSD) over Cα atoms (Cα-pRMSD) values of 0.65 and 0.62 Å on MHC-I (Fig. 2A), and 0.34 and 0.33 Å on MHC-II (Fig. 2B), respectively. Although PMGen+TE was less accurate than the IG mode, it still outperformed PANDORA and AlphaFold Multimer, achieving median Cα-pRMSD values of 1.05 Å on MHC-I and 0.42 Å on MHC-II.

We additionally report full-atom pRMSD to evaluate side-chain orientation accuracy. PMGen+pLDDT and PMGen achieved the best performance on MHC-I, both with a median full-atom pRMSD of 1.39 Å (Fig. 2C). On MHC-II, however, PMGen did not outperform Tfold, whereas PMGen+pLDDT still achieved the highest performance, with a median full-atom pRMSD of 1.12 Å (Fig. 2D).

**Figure 3:**
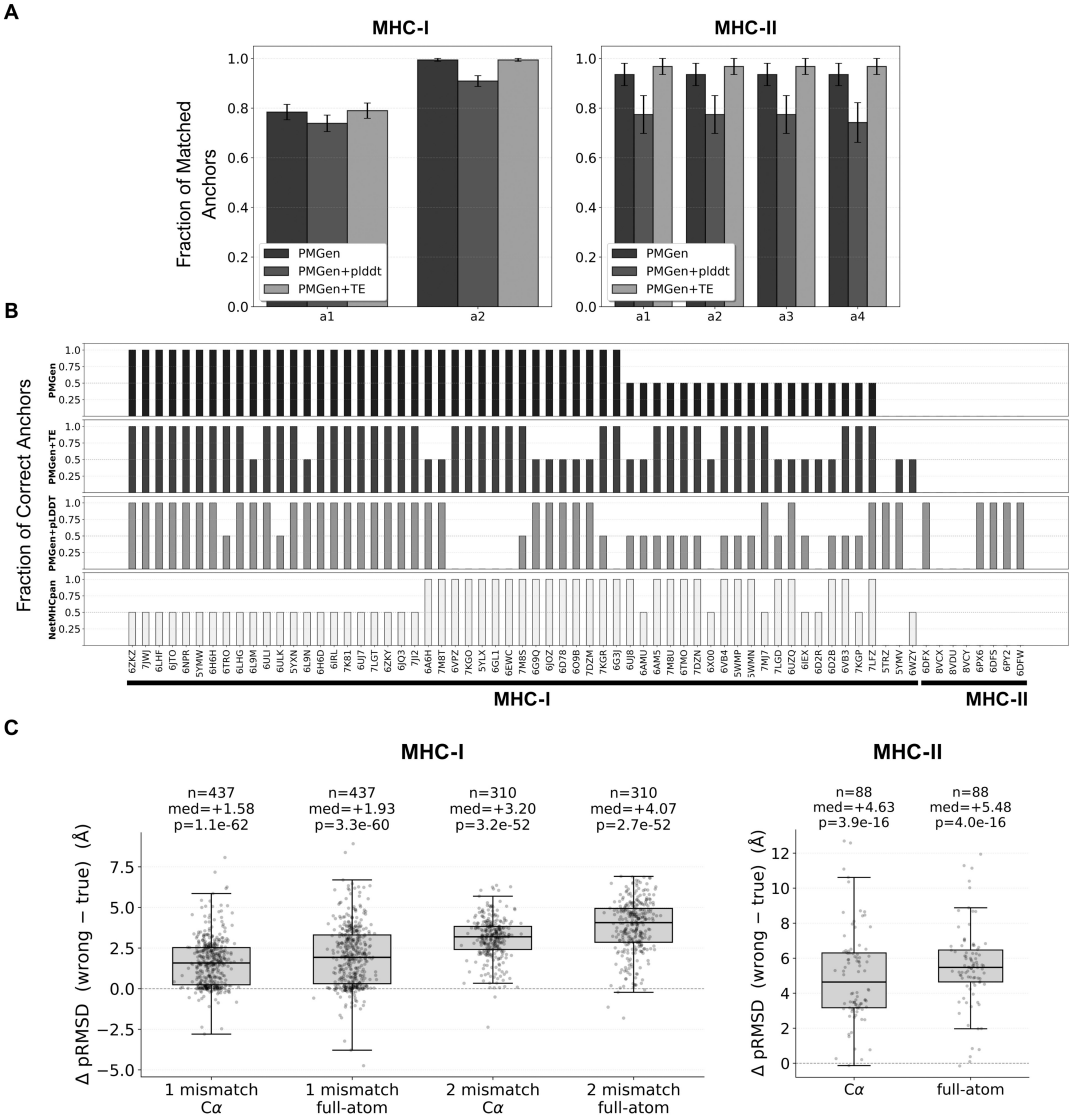
Anchor residue positioning analysis. (A) Fraction of anchors positioned that match the anchor positions predicted by NetMHCpan. (B) Structures in which at least one version of PMGen positioned an anchor differently from at least one anchor predicted by NetMHCpan. The *y*-axis shows the fraction of correctly positioned/predicted anchors relative to ground-truth structures. (C) The impact of wrong-anchor input on structure prediction is visualized for MHC-I (left) in one and two anchor mismatch scenarios, and in general on MHC-II (right). *P-*value from two-sided Wilcoxon signed-rank tests comparing paired Cα-pRMSD values of wrong-anchor and true-anchor structures for the same PDB.

**Figure 4:**
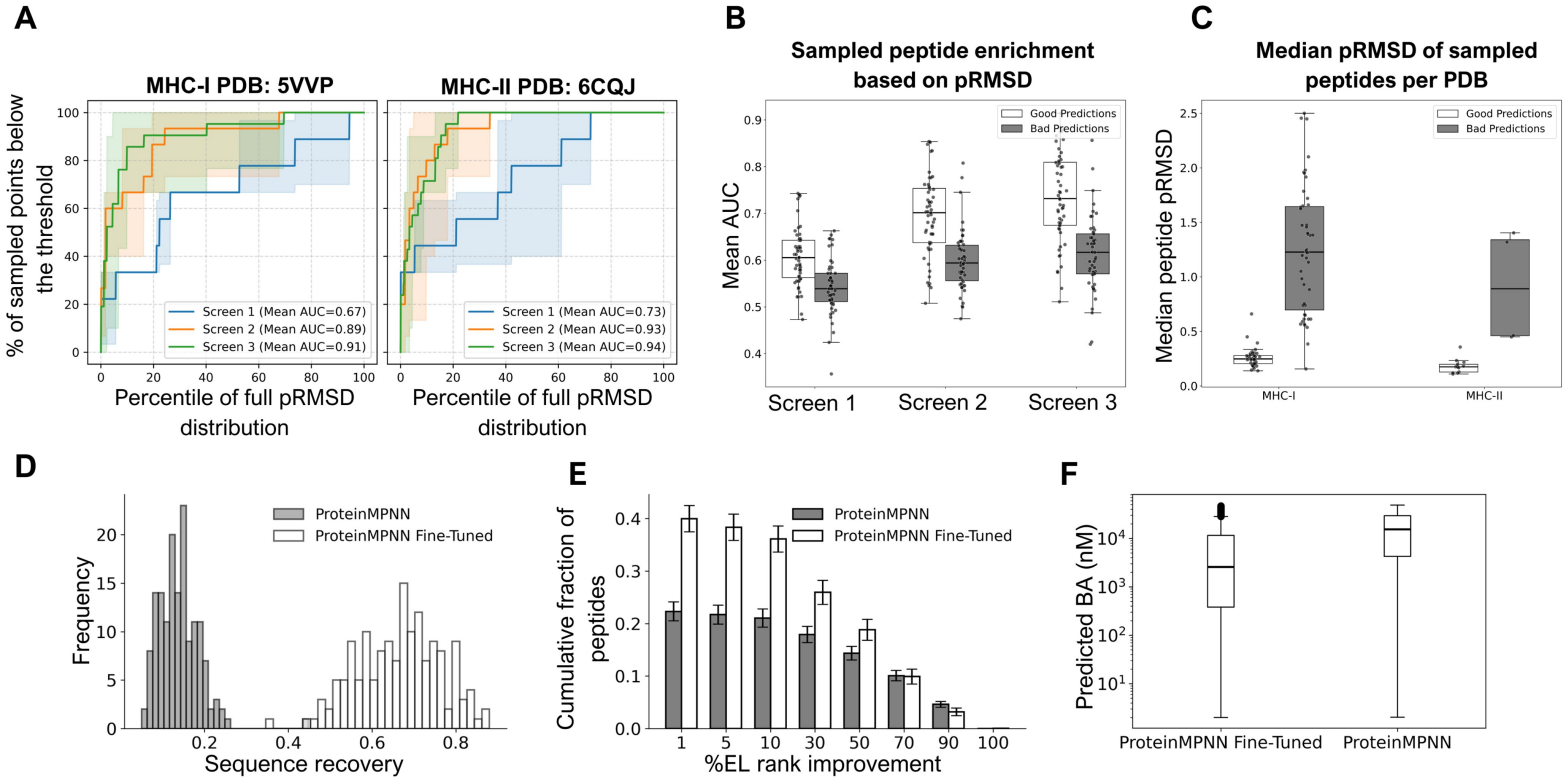
Peptide generation analysis conditioned on backbone and MHC sequence. (A) Cumulative enrichment analysis of Cα-pRMSD for PMGen-generated peptides compared to randomly mutated peptides in two sample PDBs. Screens 1, 2, and 3 correspond to sliding windows with one, two, and three point mutations, respectively. A higher area under the curve (AUC) indicates greater enrichment among peptides with lower Cα-pRMSD. (B) Comparison of Cα-pRMSD enrichment AUCs between good (low original Cα-pRMSD, n=55) and bad (high original Cα-pRMSD, n=51) predicted structures. (C) Median Cα-pRMSD values of generated peptides across all screens; each point represents the median deviation of generated peptides from the original predicted structure for a single PDB. (D) Sequence recovery distribution of sampled peptides to the original peptide. (E) Cumulative fraction of sampled peptides with improved (lower) EL percentile rank compared to the original peptide. (F) NetMHCpan-predicted binding affinities of all unique sampled peptides. Peptides were generated from the same predicted structure using the original ProteinMPNN and the version fine-tuned on PMGen structures.

Across all PMGen variants, prediction accuracy exhibited a modest negative correlation with test–train sequence similarity for MHC-I (Spearman’s ρ∈[−0.32,−0.16]). In contrast, no significant dependence was observed for MHC-II (Fig. 3 and Supplementary Methods, available as supplementary data at *Bioinformatics* online). Furthermore, when compared to other pMHC structure predictors, PMGen demonstrated the fastest computational speed for single-structure prediction (Fig. 4, available as supplementary data at *Bioinformatics* online).

### 3.3 Importance of anchor prediction in structure prediction

We evaluated the impact of anchor residue prediction on structural accuracy and assessed the concordance between the final structural anchor positions and those predicted by NetMHCpan. Previous work (Xia *et al.* 2023) characterized anchor positioning profiles, demonstrating that peptide residues with the lowest solvent-accessible surface area (SASA) strongly correlate with anchor function. Guided by this insight, we identified anchor positions for both ground-truth and predicted structures by selecting residues with minimal normalized SASA values and applying distance-based criteria (see Supplementary Methods, available as supplementary data at *Bioinformatics* online). We then performed three comparisons:

1. NetMHCpan-predicted anchors versus structurally defined anchors from predicted structures,
2. NetMHCpan-predicted anchors versus structurally defined anchors from ground-truth structures, and
3. Structurally defined anchors from predicted structures versus structurally defined anchors from ground-truth structures.

These comparisons were conducted using the benchmark dataset. For MHC-I, all PMGen variants showed lower agreement with NetMHCpan at the first anchor position, while the second anchor exhibited near-complete concordance (Fig. 3A—left). For MHC-II, agreement metrics were identical across all anchor positions (a1–a4) because the relative inter-anchor spacing is fixed; thus, a mismatch in the first anchor propagates to all subsequent positions (Fig. 3A—right). PMGen and PMGen+TE, which rely on NetMHCpan input, exhibited high agreement with NetMHCpan regarding the positioning of all four MHC-II anchors. As expected, PMGen+pLDDT, which operates without prior anchor constraints, positioned anchors differently.

**Figure 5:**
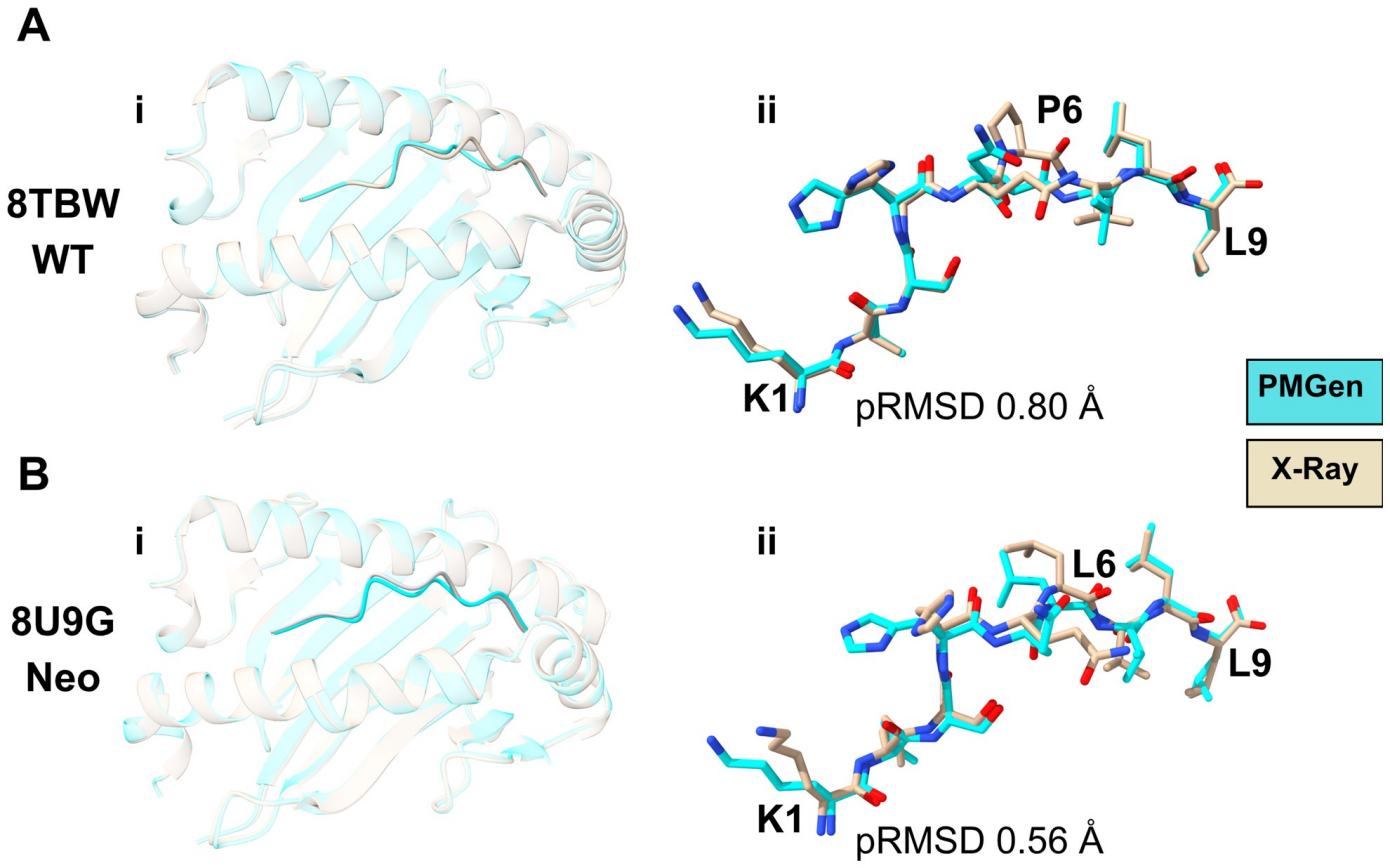
Wild-type antigen versus neoantigen modeling with PMGen. (A) Wild-type antigen KLSHQPVLL: (i) Predicted structure aligned with the X-ray structure. (ii) Peptide alignment showing that Cα atoms of almost all residues match, with the exception of P6. (B) Neoantigen KLSHQLVLL: (i) Predicted structure aligned with the X-ray structure. (ii) PMGen correctly predicts the side chain of L6 pointing outward.

We identified 59 pMHC-I and 8 pMHC-II structures where at least one PMGen variant mismatched NetMHCpan predicted anchor positions (Fig. 3B). PMGen incorrectly positioned anchors that were correctly predicted by NetMHCpan in only 13 cases. Conversely, in the majority of instances, PMGen successfully corrected anchors mispredicted by NetMHCpan that can result in Cα-pRMSD improvement (Fig. 5A-i and ii, available as supplementary data at *Bioinformatics* online). While PMGen+TE followed a similar trend, it occasionally misplaced anchors even when NetMHCpan predictions were correct (Fig. 5A-iii, available as supplementary data at *Bioinformatics* online). PMGen+pLDDT correctly recovered anchor positions in 23 cases where NetMHCpan failed for MHC-I and 5 cases for MHC-II (Fig. 3B; Fig. 5B, available as supplementary data at *Bioinformatics* online).

To test the impact of incorrect anchor input on structure prediction accuracy, we selected cases where the correct input anchor was positioned correctly in the final structure in IG mode. We then predicted the same structures with incorrect anchor inputs (1 or 2 mismatches for MHC-I, and all possible incorrect anchor combinations for MHC-II) and calculated how much this mismatch decreased performance in pRMSD. The results showed a significant drop in performance across all scenarios, indicating the importance of correct anchor input for structure prediction (Fig. 3C).

### 3.4 Applications of PMGen in structure-aware peptide design and model fine-tuning

As a secondary objective, we investigated PMGen’s applications in two fields: peptide design and generating accurate structures for training machine learning models. For the first purpose, we integrated ProteinMPNN (Dauparas *et al.* 2022) into the PMGen pipeline to enable structure-aware peptide generation for specific target MHC molecules (see Supplementary Methods, available as supplementary data at *Bioinformatics* online). For the second purpose, we fine-tuned ProteinMPNN on predicted structures from an Immune Epitope Database (IEDB, https://www.iedb.org/)-derived pMHC dataset to improve ProteinMPNN’s main objective (sequence recovery) and to enhance higher affinity peptide sampling.

For the first objective, we sampled peptides with ProteinMPNN conditioning on PMGen-predicted pMHC backbone, the corresponding MHC sequence, and any fixed (nonvariable) peptide positions. We utilized the same dataset from the structure prediction benchmark for evaluation. PMGen predictions were categorized into 51 high Cα-pRMSD (>1.0 Å) and 55 low Cα-pRMSD (<0.6 Å) structures. We performed three *in silico* screens with sliding windows of single, double, and triple consecutive amino acid substitutions. For each window, we generated peptide variants using ProteinMPNN and selected candidates based on their NetMHCpan-predicted %EL ranks. Randomly mutated peptides served as controls (see Supplementary Methods, available as supplementary data at *Bioinformatics* online, for sampling details). After predicting the structures of sampled and randomly mutated peptides, we calculated the C*α*-pRMSDs versus the original peptide’s predicted structures. Cumulative enrichment analyses were conducted for each screen and structure separately (Fig. 6, available as supplementary data at *Bioinformatics* online). We measured the enrichment of ProteinMPNN-sampled peptides relative to the random mutation baseline in terms of Cα-pRMSD (Fig. 4A). The results demonstrated higher enrichment (area under the curve) for PMGen predictions with low Cα-pRMSD (Fig. 4B). The absolute Cα-pRMSD comparison between ProteinMPNN-sampled peptides across good and bad predictions indicates five times lower deviation from the original structure in the good prediction group (Fig. 4C). These results indicate that PMGen high-quality structures, which show significantly better AlphaFold confidence metrics (Fig. 7, available as supplementary data at *Bioinformatics* online), can be used to sample peptides with similar structural properties.

For the fine-tuning application, we sampled a dataset of 163 948 pMHC binders from the IEDB database, encompassing 426 human and nonhuman MHC-I alleles. Following structure prediction, we retained only the 87 187 structures with a peptide pLDDT score above 80. To prevent dominant MHC-I alleles from biasing the dataset, we applied a median stratification sampling technique both before and after structure prediction. This yielded a final set of 63 817 structures. These were used to fine-tune ProteinMPNN utilizing an MHC-allele-based fivefold cross-validation split, alongside a separate test set comprising the 20% rarest alleles (see Supplementary Methods and Fig. 8A–E, available as supplementary data at *Bioinformatics* online). We fine-tuned ProteinMPNN using sequence recovery, consistent with its original training objective (Fig. 8F, available as supplementary data at *Bioinformatics* online). The performance was evaluated for each fold on the test set (Fig. 8G, available as supplementary data at *Bioinformatics* online). We sampled peptides from the test set using both ProteinMPNN and its fine-tuned version for comparison. Fine-tuning increased peptide sequence recovery on the test set from a mean of 0.14–0.64 (Fig. 4D). We also observed that at least 40% of sampled peptides from the fine-tuned model had lower predicted eluted ligand (EL) percentile rank than the original peptide, compared to 23% for the baseline ProteinMPNN (Fig. 4E), with a seven-fold improvement in mean predicted binding affinity (Fig. 4F).

### 3.5 PMGen evaluation on a neoantigen/wild-type pair test case

To demonstrate PMGen’s capability in distinguishing subtle structural differences between antigens, we evaluated its performance on a wild-type/neoantigen pair previously used to benchmark Tfold (Mikhaylov *et al.* 2024). Specifically, we modeled the wild-type antigen KLSHQ**P**VLL bound to HLA-A*02:01 (PDB: 8TBW) and its single-point mutation neoantigen variant KLSHQ**L**VLL (PDB: 8U9G). PMGen was executed in IG mode for both cases, and the predicted models were compared to the experimental X-ray structures.

For the wild-type complex, PMGen achieved a Cα-pRMSD of 0.80 Å (Fig. 5A), a notable improvement over the previously reported Tfold Cα-pRMSD of 1.2 Å. The predicted peptide orientation and anchor positioning closely resembled the crystal structure (Fig. 5A-i). Residues K1, L2, S3, L8, and L9 were closely aligned with the experimental coordinates, while P6 and V7 showed a minor shift in their Cα positions (Fig. 5A-ii). Side-chain rotamers for H4 and Q5 showed minor deviations, though the backbone alignment was largely preserved.

For the neoantigen (P6→L6), PMGen achieved a Cα-pRMSD of 0.56 Å (Fig. 5B-i), performing comparably to Tfold (0.6 Å), with Cα positions closely aligned to the reference structure. In the experimental structure, the side chain of the mutated L6 is oriented outward and slightly toward an MHC α-helix. PMGen correctly predicted this orientation. Similar to the wild-type case, the predicted rotamers for H4 and Q5 differed slightly from the experimental structure (Fig. 5B-ii).

## 4 Discussion

PMGen is a versatile framework for pMHC structure prediction and structure-guided peptide design, integrating anchor residue information into AlphaFold2 (Jumper *et al.* 2021) via two modes: IG and TE. Unlike existing tools such as MHC-Fine (Glukhov *et al.* 2024), which are limited to MHC-I and short peptides, PMGen supports both MHC classes and a broad range of peptide lengths.

When benchmarked against state-of-the-art methods (Evans *et al.* 2021, Marzella *et al.* 2022, Parizi *et al.* 2023, Glukhov *et al.* 2024, Mikhaylov *et al.* 2024), both PMGen and its anchor-blind variant, PMGen+pLDDT, demonstrated superior structure prediction accuracy (Fig. 2). Additionally, PMGen outperformed competitors in single-structure prediction speed on a GPU, owing to the elimination of time-consuming multiple sequence alignment (MSA) searches and the use of a smaller template database. To ensure a fair comparison, we explicitly excluded overlapping templates and AlphaFold training structures during the PMGen runs. However, because we lacked full control over the pipelines of the competing tools, it is possible that some models benefited from information leakage via MSAs, structural templates, or validation set overlaps. Furthermore, we observed that sequence similarity to the AlphaFold training set had a negligible impact on test set performance for MHC-I, and no observable impact for MHC-II (Fig. 3, available as supplementary data at *Bioinformatics* online). While these results are highly encouraging, the small sample size of the MHC-II test set (n=31) remains a notable limitation.

Our results demonstrate that AlphaFold effectively assimilates anchor information from both IG and TE modes without requiring parameter fine-tuning, with IG mode being the superior. This inherent flexibility aligns with recent findings that AlphaFold can leverage contact maps and distance constraints to resolve structural ambiguities (Stahl *et al.* 2023, 2024). Furthermore, the utility of providing multiple structural priors to enhance prediction and design is well documented (Bennett *et al.* 2023, Bradley, 2023). Remarkably, we observed that the IG strategy outperformed TE (Fig. 2). We hypothesize that this improvement stems from how AlphaFold processes conflicting template data. When presented with multiple similar templates, the model tends to converge on an average consensus conformation with artificially high confidence. This behavior restricts the search space to the close neighborhood of the engineered templates. In contrast, the IG mode provides only coarse spatial constraints relative to the MHC. This looser guidance enables AlphaFold to explore a broader conformational landscape.

PMGen profits from being provided with correct anchor positions (Fig. 3A and C), yet it demonstrates some capacity to recover correct positions even when anchor inputs are wrong (Fig. 3B). This highlights AlphaFold’s inherent denoising capability regarding partial conformations (Gut and Lemmin 2025). Since different PMGen modes yield distinct structural ensembles, their combination increases the probability of sampling the native conformation. Crucially, we also observed that correct anchor positioning correlates strongly with higher pLDDT scores (Fig. 7, available as supplementary data at *Bioinformatics* online) (Mikhaylov *et al.* 2024). The practical value of this metric was evident in five challenging MHC-II cases where only PMGen+pLDDT resolved the correct core (Fig. 3B; Fig. 5B, available as supplementary data at *Bioinformatics* online). These findings show that structural confidence scores can reduce errors in sequence-based anchor prediction.

PMGen’s accurate structures enable translational applications, such as structure-aware peptide generation to profile MHC binders and providing training data for structure-based machine learning models. To this end, we integrated ProteinMPNN (Dauparas *et al.* 2022) into our workflow to leverage its autoregressive sampling. We used NetMHCpan (Nilsson *et al.* 2025) to rank sampled candidates in the current paper. This choice was motivated by its broad allele coverage (Atkins *et al.* 2023). Our enrichment analysis revealed that generated peptides preserve the structure of the original peptide. This effect was especially pronounced in screens with a higher number of mutations (Fig. 4A and B). We showed that high structure preservation is achieved with high-quality predicted structures (Fig. 4C). As a second application, we showed that PMGen-predicted structures can be used to train machine learning models. In the current study, we fine-tuned ProteinMPNN as an example. This successfully improved average peptide sequence recovery from 0.14 to 0.64 when used for peptide sampling from rare HLA alleles (Fig. 4D). As an indirect metric, we observed that upon fine-tuning the fraction of peptides with higher predicted affinity also increased (Fig. 4E and F). While in the current study we focused on binding affinity, *in silico* metrics can be optimized for specific goals, such as TCR-specific rankings (Dolton *et al.* 2023). These findings emphasize the possible applications of PMGen high-quality predicted structures in vaccine design and machine learning fields.

Beyond these computational applications, PMGen’s structural accuracy also has direct implications for neoantigen discovery and optimization. Our method addresses critical limitations in current neoantigen prediction tools, which often rely on sequence-based features or low-resolution structural models (Kim *et al.* 2023, Wang *et al.* 2023). By providing high-quality 3D structures and enabling structure-aware generation, PMGen facilitates the rational design of immunogenic neoantigens and enhanced mimotopes (Visani *et al.* 2025). In a benchmark case, PMGen accurately captured mutation-induced conformational changes that are critical for altered T-cell recognition (Fig. 5) (Zhang *et al.* 2024, Yuan *et al.* 2025). Such mimotope-based strategies have shown promise in preclinical models and early phase clinical trials (Ribas *et al.* 2011, Buhrman and Slansky 2013, He *et al.* 2022, Martínez-Cortés *et al.* 2023, Zhou *et al.* 2024). They also offer similar therapeutic potential in autoimmune diseases (Song *et al.* 2023). PMGen can offer faster alternatives to MD-based frameworks by screening over the structure of thousands of candidate peptides (Zhong *et al.* 2025). Recent work demonstrates that incorporating 3D MHC-II coordinates yields experimentally validated neoantigens (Yuan *et al.* 2025). PMGen’s dual-class support extends this paradigm. This capability is particularly important, as combining MHC-I and MHC-II epitopes enhances therapeutic efficacy (Roerden and Spranger 2025). While the current study primarily establishes the PMGen framework, its direct application to clinical vaccine design remains a promising avenue for future investigation.

Several limitations of the current study should be acknowledged. First, PMGen relies on AlphaFold2. While our anchor-constraint strategy substantially improves peptide docking performance, newer architectures like AlphaFold3 (Abramson *et al.* 2024) may offer further enhancements. However, AlphaFold3’s recent training cutoff complicates unbiased benchmarking against existing methods. Second, the default anchor prediction depends on NetMHCpan. This tool may underperform for rare or poorly characterized alleles, though the pLDDT-based selection mode provides a sequence-independent fallback. Third, our peptide generation module shows encouraging *in silico* enrichment for predicted binding affinity. However, these candidates have not yet been experimentally validated. Predicted binding affinity is a necessary but insufficient proxy for immunogenicity. This property additionally depends on antigen processing, peptide stability, and the TCR repertoire. Finally, the current design module optimizes for MHC binding without explicitly modeling TCR interactions. This constraint could be addressed through integration with emerging TCR-pMHC modeling approaches (Bradley 2023).

PMGen offers a robust and versatile framework for pMHC modeling and neoantigen design. The pipeline is well suited for integration into both experimental workflows for cancer immunotherapy and autoimmune research, as well as structure-based *in silico* pipelines. By enabling the high-throughput generation of accurate structural models, PMGen serves as a critical first step in producing high-quality synthetic training data for machine learning frameworks, directly addressing the limitations and biases inherent in current experimental and computational datasets. Our future work will focus on the *in vitro* and *in vivo* validation of PMGen-derived neoantigens, improved model interpretability, and integration with advanced generative architectures (Deng *et al.* 2024, Lin *et al.* 2024) to accelerate the development of next-generation immunotherapies.

## Supplementary Material

btag381_Supplementary_Data

## Data Availability

The datasets used for benchmarking and fine-tuning are available from the IEDB database and PDB as described in the Materials and methods section. All the generated structures are available at https://wwwuser.gwdguser.de/compbiol/pmgen/pdbs. The source code for PMGen is available on GitHub https://github.com/soedinglab/PMGen repository. The code and data to reproduce paper results is available at https://wwwuser.gwdguser.de/compbiol/pmgen/. A user-friendly notebook to run PMGen is available at https://colab.research.google.com/github/soedinglab/PMGen/blob/master/colab.ipynb. The code developed to fine-tune ProteinMPNN, including model weights is available at https://github.com/AmirAsgary/ProteinMPNN_FineTune.
