# Supplementary material for: PMGen: from peptide-MHC structure prediction to peptide generation
Source: Bioinformatics. 2026 Jun 15;42(6):btag381. doi: 10.1093/bioinformatics/btag381 (PMC13308714; doi:10.1093/bioinformatics/btag381)
Supplement: btag381_Supplementary_Data [file btag381_supplementary_data.pdf]

Supplementary Methods

PMGen: From Peptide-MHC Structure Prediction to Peptide Generation

Binding Groove Distance Distribution

We selected the longest sequences among the processed structures (PDB ID: 4U6Y for MHC-I, 4H25 for MHC-II). We then manually defined the right, core, and left regions on the α structures. The right region was identified as the set of amino acids $\{4,5,6\}$ following the nearest MHC_α residue to the first tightly bound peptide amino acid (with a C_α distance < 4.5 Å). The left region was defined using the same rule but on the opposite side, primarily where long peptides protrude from MHC-II. The core region was considered a broader segment between the right and left regions, consisting of 10 amino acids. For each region, the nearest partner amino acids on the opposing MHC_α secondary structure were identified based on their sequence position and the middle amino acid's Euclidean distance. These distances were averaged for each side. For the remaining structures, we determined the right, core, and left regions by aligning their amino acids to those of the selected structures (Figure S1). Alignments were performed using Clustal Omega multiple sequence alignments (MSAs) (Sievers et al., 2011).

AlphaFold Prediction

To predict final structures, we used the Python implementation of AlphaFold (Motmaen et al., 2023).This implementation is based on the AlphaFold2 architecture and adapted for multi-chain prediction.

Multi-chain functionality is enabled by shifting the residue indices of each chain by 200. This prevents AlphaFold from interpreting separate chains as a continuous polypeptide. For each prediction, the query sequence was aligned to the engineered templates and supplied to AlphaFold.

The number of recycling iterations in AlphaFold was kept at the default value of three. For benchmarking, we used AlphaFold's `model_2_ptm` as it showed the highest confidence scores for pMHC structure prediction without MSA inputs (Figure S2).

Structure Prediction Benchmarking Setting

For benchmarking with AlphaFold-Multimer 2.2, we used a local installation of ColabFold v1.5.5 (Mirdita et al., 2022). Multiple sequence alignments and template searches were performed against the UniRef30 and PDB70 databases, respectively. Predictions were generated using the five default AlphaFold models, and only the top-ranked structure reported by AlphaFold was used in the benchmarking analysis.

Tfold (Mikhaylov et al., 2024) was executed with default settings, using its integrated SeqNN model for anchor prediction. As Tfold outputs multiple ranked structures, only the top-ranked structure generated by the tool was considered for benchmarking.

PANDORA (Marzella et al., 2022) predictions were performed with default settings, using the built-in PANDORA BLAST and PDB databases for homology search and template selection. To automate anchor position assignment, we enabled the NetMHCpan anchor prediction option (Reynisson et al., 2020).

MHC-Fine (Glukhov et al., 2024) was executed locally with default settings. MSA generation involved jackhmmer queries to its dedicated database for MHC complexes.

For PMGen and PANDORA benchmarking, we applied a leave-one-out strategy during template search and engineering. Specifically, templates with the same PDB ID as the target structure were excluded to avoid potential bias. In addition, we only allowed structures released before each benchmarking case to be used as templates in PMGen. We did not do this for PANDORA, as it required violation in their original code.

For Multimer 2.2, Tfold, and MHC-Fine, template searches were executed internally by the tools themselves. Because these methods lack a built-in leave-one-out benchmarking protocol, identical template sequences were not explicitly excluded. Furthermore, we utilized their default MSA search and input modules, introducing a potential avenue for information leakage. An additional source of overlap stems from MHC-Fine, which used nearly half of our benchmarking dataset for validation. Given the scarcity of available benchmarking data, we opted not to exclude these structures. Consequently, to mitigate data leakage for these competing methods, we relied exclusively on the strict training structure cut-off date.

All tools were benchmarked on both MHC-I and MHC-II, except for MHC-Fine, which only supports MHC-I. To ensure fairness, deviations from the default tool settings were minimized. For AlphaFold-based methods, parameters with a training cut-off of 2018 were used by default. All tools were benchmarked on PDB structures released after this training cut-off.

Structure Prediction Evaluation Metrics

To compare structure prediction models, the primary evaluation metric was the root-mean-square deviation (RMSD) between the predicted and true structures of a peptide i with length L, after superposition based on the MHC structures. We report two variants: the Cα-pRMSD, computed over only the Cα atoms,

$$\mathrm{C}\alpha\text{-pRMSD}_i = \sqrt{\frac{1}{L}\sum_{j=1}^{L}\left\|\mathbf{r}_{i,j}^{\mathrm{CA,pred}} - \mathbf{r}_{i,j}^{\mathrm{CA,true}}\right\|^2},$$

and the full-atom pRMSD, computed over all N_i non-hydrogen atoms of the peptide,

$$\text{Full atom -pRMSD}_i = \sqrt{\frac{1}{N_i}\sum_{k=1}^{N_i}\left\|\mathbf{r}_{i,k}^{\mathrm{pred}} - \mathbf{r}_{i,k}^{\mathrm{true}}\right\|^2}.$$

The predicted aligned error (PAE) was symmetrized for each residue pair using

$$\mathrm{PAE}_{ij}^{\mathrm{sym}} = \frac{\mathrm{PAE}_{ij} + \mathrm{PAE}_{ji}}{2}.$$

Only amino acid pairs within a 10 Å radius were considered when computing the final average per-residue PAE. This value is reported and used throughout the current study. The predicted lDDT scores provided directly by AlphaFold 2 were also used as an evaluation metric.

Similarity-stratified performance analysis

To assess how prediction accuracy depends on the proximity of test cases to the training set, each test pMHC was assigned a similarity score defined as the maximum, over all training-set pMHCs, of

$$\mathrm{sim}_{\mathrm{pMHC}} = \frac{n_{\mathrm{MHC}}^{\mathrm{aligned}} + n_{\mathrm{peptide}}^{\mathrm{aligned}}}{n_{\mathrm{MHC}}^{\mathrm{total}} + n_{\mathrm{peptide}}^{\mathrm{total}}},$$

where the numerator is the number of identical aligned residues in the MHC and peptide chains and the denominator their combined lengths. Test cases were binned into six similarity intervals (0.65–0.85, 0.85–0.9, 0.9–0.95, 0.95–0.97, 0.97–0.99, 0.99–1); because MHC sequences are themselves highly homologous, almost all test cases fall above 0.9, and finer-grained binning in this upper range is required to resolve meaningful variation in test–train similarity. The per-bin distribution of Cα- and full-atom pRMSDs is visualised in Figure S3 for each combination of MHC class, method, and structural representation.

To quantify the strength of this dependence, we computed Spearman's rank correlation ρ between $\mathrm{sim}_{\mathrm{pMHC}}$ and Cα-pRMSD, separately for each of the 12 strata (MHC class $\times$ method $\times$ representation), and Bonferroni-corrected the resulting p-values for the 12 comparisons. For MHC I ($n = 176$ per stratum), PMGen and PMGen+pLDDT showed a significant negative correlation under both representations (PMGen: $\rho = -0.30$ Cα, $\rho = -0.32$ full-atom; PMGen+pLDDT: $\rho = -0.28$ Cα, $\rho = -0.29$ full-atom; all Bonferroni-corrected $p < 0.01$). PMGen+TE exhibited a weaker trend ($\rho = -0.16$ Cα, $\rho = -0.20$ full-atom) that did not survive correction (Bonferroni-corrected $p = 0.36$ and $p = 0.09$ respectively), indicating that its accuracy tracks training-set similarity less consistently than the other two variants. The corresponding endpoint comparison—median Cα-pRMSD in the lowest- versus highest-similarity bin (0.65–0.85 vs 0.99–1)—was, for Cα-pRMSD, 0.74 vs 0.44 Å for PMGen, 1.34 vs 0.83 Å for PMGen+TE, and 0.74 vs 0.41 Å for PMGen+pLDDT; and for full-atom pRMSD, 1.78 vs 1.20 Å for PMGen, 2.05 vs 1.58 Å for PMGen+TE, and 1.23 vs 1.16 Å for PMGen+pLDDT. For MHC II ($n = 31$ per stratum), point estimates of ρ were directionally consistent with the MHC I trend ($\rho \in [-0.21, -0.13]$ for PMGen and PMGen+TE under both representations) but no stratum reached significance after correction, which we attribute to the limited sample size.

Speed Benchmark

Prediction speeds were evaluated in single-run mode using the pMHC-I structures from our benchmarking set, with execution times averaged across all successful predictions. To isolate pure inference speed, we excluded AlphaFold compilation and model-loading times, defining model speed as the total execution time from the start of the prediction pipeline to the final saved results. Certain methods, such as Tfold and PMGen+pLDDT, automatically generate multiple predictions to select the best final structure; for these tools, the entire multi-step process was timed as a single, continuous run. For PANDORA, MHC-Fine, PMGen+TE, and the default PMGen mode, speed was recorded based on a single structural prediction. With the exception of PANDORA, which operates solely on a CPU, all methods were executed on an NVIDIA A100 GPU utilizing 32 GB of system RAM. Finally, although each method (including PMGen) supports parallelization, these capabilities were not utilized in the current speed benchmark to ensure a standardized comparison of baseline efficiency (Figure S4).

Defining Anchor Residues from Structure

To identify anchor residues in each predicted pMHC structure, we used the solvent accessible surface area (SASA) as a selection criterion. SASA has been shown to be a reliable structural indicator for identifying

peptide anchor positions (Xia et al., 2023). We calculated the SASA for each residue as the total solvent-accessible surface area of its atoms, using the FreeSASA package (Mitternacht, 2016). Since amino acids inherently have different range of SASA values, we normalized the total surface area by maximum allowed solvent accessibilities for each amino acid (Tien et al., 2013).

Anchor positions were determined directly from the pdb structures rather than from sequence conservation, ensuring that the definition was minimally affected by sequence identity. Our method selects residues that (i) have low SASA values and (ii) satisfy a minimum separation in sequence index (difference between their positions in the peptide sequence).

Let P be the set of all peptide residue positions in a given peptide–MHC complex. We define F as the set of sets S of all feasible anchor position combinations:

$$F = \left\{ S \subseteq P \,\middle|\, |S| = m, \forall p \in S : \quad SASA_p < 50, \quad \text{and} \quad \max(S) - \min(S) > n \right\}$$

The selected anchor residues A are then given by:

$$A = \arg\min_{S \in F} \sum_{p \in S} SASA_p \tag{S1}$$

$$\text{with} \quad \begin{cases} m = 2, & n = 6, \quad \text{if MHC-I} \\ m = 4, & n = 8, \quad \text{if MHC-II} \end{cases}$$

Here, m denotes the total number of anchor residues to be selected, and n is the minimum sequence index distance allowed between two consecutive anchors. For MHC-II, we also check if the space between each consecutive anchor is at least 2. The residues in A are therefore those that satisfy the SASA and spacing constraints while minimizing the total SASA sum.

Structural Evaluation and Accuracy Metrics

To evaluate the accuracy of the predicted pMHC structures, we first aligned the modeled MHC chains to their corresponding ground-truth reference structures. This superposition was calculated exclusively using the MHC chains to ensure the alignment was driven by the stable MHC backbone. For MHC-II complexes, both chains were concatenated and treated as a single entity during alignment to preserve their relative spatial orientation. The optimal rotation matrix derived from this MHC alignment was then applied to the predicted peptide coordinates, effectively placing the modeled peptide into the same reference frame as the ground-truth complex.

We defined the peptide core region as the sequence segment flanked by the first and last anchor residues, which were identified independently for both the predicted and ground-truth structures. To quantify structural fidelity, we computed the RMSD for both the peptide Cα atoms and full-atom peptide coordinates strictly within this core region.

Finally, to assess the accuracy of anchor positioning, we compared the sequential index of each anchor in the predicted structure against its corresponding index in the ground truth (e.g., comparing the index of anchor 1 in the prediction to anchor 1 in the reference). An anchor was considered correctly positioned only if its indices matched exactly. We applied this identical matching criterion to evaluate the concordance between NetMHCpan-predicted anchors and those found in either the predicted or ground-truth structures. The overall anchor positioning accuracy was calculated as the fraction of matched anchors:

$$\text{Fraction of Matched Anchors} = \frac{\text{Number of matched anchors}}{\text{Total number of anchors}} \tag{S2}$$

ProteinMPNN Integration

We used ProteinMPNN (Dauparas et al., 2022) to sample new peptide binders for a given pMHC backbone structure. In ProteinMPNN, the encoder receives the structure as input rather than the sequence. The decoder can generate sequences either by conditioning on both the structure and existing sequence information (conditional mode) or by using only the encoded backbone (unconditional mode). We applied the conditional mode in this study, as the backbone of MHCs is highly conserved and does not provide sufficient information for peptide sampling across different alleles.

In conditional mode, we distinguish two types of amino acid positions in the structure:

- **Unfixed (designable or variable)**: Amino acid positions whose identities are not provided to the model; the decoder samples new residues for these sites.
- **Fixed (non-designable or conserved)**: Amino acid positions whose identities are provided to the model as conditioning information (along with the backbone) and are therefore not altered during sequence generation.

In PMGen, the MHC sequence is always fixed while the peptide sequence is sampled. By user's choice, the peptide's anchor positions can be fixed. The fraction of randomly fixed non-anchor peptide positions can also be adjusted by the user to control the level of design flexibility.

NetMHCpan Integration

In the current pipeline, we used NetMHCpan 4.1 (Reynisson et al., 2020) for MHC-I and NetMHCIIpan 4.3 (`https://services.healthtech.dtu.dk/services/NetMHCIIpan-4.3/`) (DTU Health Tech, 2025) for MHC-II. NetMHCpan is applied twice during the PMGen workflow: (i) for anchor prediction prior to PANDORA homology modeling, and (ii) after ProteinMPNN peptide generation to rank candidate peptides and select binders based on their predicted binding affinity (BA) and EL percentile rank.

Since NetMHCpan requires an HLA allele and peptide sequence as input, PMGen first searches for compatible alleles using a sequence alignment against known HLA alleles. Once a matching allele is identified, PMGen checks whether it is supported by NetMHCpan. If not, the most similar NetMHCpan-accepted allele is selected based on sequence similarity.

The PMGen pipeline supports two modes of anchor prediction:

1. **Multiple-anchor prediction**: NetMHCpan is run across varying peptide lengths (if the peptide is longer than 8 amino acids). Predicted anchor positions are ranked by EL percentile rank and, secondarily, by predicted binding affinity. The top k anchors (where k is user-defined) are then selected. For each selected anchor, PMGen predicts a distinct peptide–MHC structure. The best AlphaFold-predicted structure is chosen based on its average peptide-pLDDT score.
2. **Single most-reliable anchor**: Only the highest-ranked anchor predicted by NetMHCpan is used, and a single structure is predicted for the peptide–MHC pair.

In the benchmarking experiments reported in this paper, the second mode was used for PMGen initial guess and PMGen+TE, while for PMGen+pLDDT we tested all possible anchor combinations and selected the best based on pLDDT.

Peptide Generation Analysis

The PDB structures predicted by PMGen were categorized into two groups based on their structural accuracy: high-quality predictions ($n = 55$, Cα-pRMSD < 0.6) and low-quality predictions ($n = 51$, Cα-pRMSD > 1.0). Three mutation screens were designed, in which one (screen 1), two (screen 2), or three (screen 3) amino acid positions were treated as variable, while the remaining residues were fixed. The variable residues were defined as contiguous linear segments within the peptide sequence.

For each structure and each screen, ten peptide variants were sampled using ProteinMPNN. Their eluted ligand (EL) percentile ranks were predicted using NetMHCpan, and the top three peptides with the lowest ranks were selected. As a control, all possible $20^k \times \mathrm{L}_{\mathrm{peptide}}$ random mutations for screen k were generated. Since the number of random mutations was prohibitively large, we randomly selected 1% of peptides. We ensured that all mutation windows and all screens across all structures and PDB ids have relatively equal fraction of samples. In total, we analyzed 29,802 low Cα-pRMSD (good predictions) and 27,161 high Cα-pRMSD (bad predictions) cases along with ProteinMPNN-sampled peptides. We then calculated their Cα-pRMSD versus the original peptide's predicted structure.

Finally, for each mutation window, enrichment analyses were performed comparing Cα-pRMSD distributions of sampled peptides against those of random peptides. The area under the curve (AUC) was computed for each mutation window and subsequently averaged across all windows corresponding to each pMHC structure (Figure S6).

Fine-tuning ProteinMPNN

Data preparation ProteinMPNN is the most widely used model for protein design tasks. However, similar to AlphaFold, it lacks sufficient observations of stable peptide–MHC structures in its training data. In the current study, we used the PMGen pipeline to generate high-quality pMHC structures to fine-tune ProteinMPNN. For this purpose, we first downloaded eluted ligand and binding affinity data from IEDB. To reduce training noise arising from anchor complexity, we used only MHC-I data for training; however, the same methodology is also applicable to MHC-II. We removed all pMHC pairs labeled as non-binders ($\mathrm{EL}_{label} = 0$ or $\mathrm{BA} > 500\,\mathrm{nM}$).

To account for MHC diversity, we counted the number of times each MHC allele was observed in combination with different peptides. The resulting per-allele counts were strongly right-skewed, with a few alleles contributing orders of magnitude more samples than the majority. We then calculated the median of these counts across all sampled MHC alleles and randomly sampled that number of pMHC pairs for each allele, iteratively. All MHC alleles with counts below the median were fully sampled and removed from the next sampling iteration; alleles whose accumulated draws already met or exceeded the new target were likewise retired. We continued this procedure with a sampling cap of 1000 samples per allele, meaning that once an allele reached 1000 samples, it was automatically excluded from subsequent iterations. Because the median is recomputed after each retirement, the target rises as smaller alleles drop out, while highly abundant alleles are progressively capped at a level set by the dataset itself rather than by a fixed quota. This method ensured that MHC alleles with fewer samples were fully represented, while highly abundant alleles were capped, allowing us to construct a well-distributed MHC dataset.

We then predicted structures for the MHC-allele-balanced samples using PMGen in anchor blind initial guess mode (PMGen+pLDDT). For each peptide–MHC pair, anchor combinations were enumerated without relying on NetMHCpan, restricting to combinations where the first anchor is at position P1 or P2 and the last anchor at the C-terminal residue, consistent with known MHC-I binding constraints. Two structures were generated per pair. We retained the one with higher mean peptide pLDDT and excluded samples with mean peptide pLDDT below 80, yielding 87,187 high-confidence structures spanning 426 MHC-I alleles (Figure S8A and B). To further correct residual MHC allele bias among the predicted structures, we re-applied iterative median sampling, resulting in a final dataset of 63,817 structures (Figure S8C). After pLDDT filtering, no allele retained more than 876 samples, so the 1000-sample cap was never reached during this second round of sampling. Instead, the effective cap emerged from the iterative median procedure itself: once the lower-count alleles had been fully sampled and retired in the first iterations, the remaining active alleles all had comparably large pools and similar accumulated draws, so the next recomputed median fell below their current tallies and they retired simultaneously. This produced a data-driven cap at 376 samples per allele, applied to roughly the top quartile of alleles, while the remaining 75% were retained in full.

Training and inference Data splitting was performed based on MHC alleles which is the standard way to split pMHC data (Wongklaew et al., 2024). Alleles were ranked by frequency and the rarest 20% (85 alleles) were held out as an independent test set, while the remaining alleles were divided into 5 folds for cross-validation (Figure S8C-E).

During training, the default temperature of 1 was used to evaluate the performance for training and validation set (Figure S8F). The same setting was applied for test set single prediction run to evaluate sequence recovery/unmasking performance (Figure S8G). For sampling runs used in combination with PMGen, we used temperature of 1.5 to have higher diversity in sequences of sampled peptides. The same temperature was also used for original ProteinMPNN parameters for fair comparison with the fine-tuned version.

We did not modify the ProteinMPNN architecture, and fine-tuning used the original objective: autoregressive prediction of masked amino acids in a random decoding order, conditioned on the full structure. Peptide residues were always masked and served as the primary prediction target. To encourage the model to learn peptide–MHC interaction properties, we additionally masked 10% of the MHC tokens at each iteration and included them in the objective; the remaining MHC residues stayed fixed as conditioning context.

To minimize deviation from, or unlearning of, the original ProteinMPNN model, we employed two adaptation strategies: elastic weight consolidation (EWC) and low-rank adaptation (LoRA). We additionally explored several hyperparameters, including the learning rate and the number of frozen layers. Training was carried out using a masked negative log-likelihood (categorical cross-entropy) loss. Peptide sequence recovery was used as the primary evaluation metric. Our results indicated that LoRA, without freezing any layers and using a learning rate of $1e-9$, yielded the optimal configuration.

Main Changes in PANDORA Pipeline

To enable PANDORA to operate robustly in the best-anchor-predicted mode, we modified its built-in NetMHCpan prediction logic. In the original implementation, only the best-aligned allele was used for anchor prediction. In our modified version, the top 20 aligned alleles are selected and ranked by alignment score. NetMHCpan is then executed iteratively, starting from the highest-ranked MHC sequence. If the NetMHCpan prediction is successful (i.e., the allele is present in the NetMHCpan's `allelelist.txt` file),

the loop terminates and that prediction is used to determine the anchor positions.

This modification improves anchor assignment for MHC queries with low sequence similarity to known alleles, as it allows the algorithm to fall back to progressively less similar alleles until a valid prediction is found.

Additionally, we made minor changes to the `Modeling_Functions.py`, `PMHC.py`, and main `PANDORA.py` scripts to:

- fix bugs affecting NetMHCpan execution,
- modify output parsing for the PMGen pipeline, and
- save predicted anchors and alignment results as `.json` files.

Further details on these modifications are available on our GitHub repository: `AFfine_PANDORA_modifications.txt`.

Supplementary Tables and Figures

Table S1: Excluded PDBs for benchmarking

Exclusion reason	PDB IDs
Broken chain	6XC9, 6XCO, 6XCP, 6Z9X, 7ALO, 6VQ2, 6WL3, 6L9K, 6L9L, 6JP3, 6PBH, 6D2B
Long peptide	6BGA
Short peptide	9B7B, 6T3Y, 6MNG, 8TNJ, 7LFI, 7LFJ, 7LFK, 7LFL, 7LFM, 7BYD, 6VQD, 6VQE, 6VQY, 6VQZ, 7KGT, 6LAM, 6LAH, 6LB2, 6LT6, 6TDO, 6TDP, 6TDQ, 6TDR, 6TDS, 6IWG, 6IWH, 6GB5, 6GB6, 6GB7, 6MP1
MHC-like structure	8T4Z, 6WL2, 6V7Y, 6V7Z, 6W9U, 6W9V, 6OMG, 6PUF, 6QIO, 6QIP, 6NUX, 6NHA, 6MWR, 6MIY, 6FGB
Non-standard amino acid	5NIG, 6V0Y
Redundant / duplicate	6V13, 6V15, 6V18, 6V19, 6V1A, 6P27, 6P2S, 6P23, 6EQA, 6EQB, 6ILE, 5NHT, 5NQK, 5WMQ, 6MTM, 6JTP, 6ULR, 6ULN, 6JTN, 6UON, 8VDD, 6Y27, 6Y28, 6Y2B, 6VRM, 6VR5, 6VRN, 6VM8, 6BJ2, 6VMA, 6G9R, 5YXU, 6P64, 6UK4, 6UK2, 6PZ5, 6PYW, 6V3J, 6AMT, 6KWK, 7F4W, 6GH1, 7N1B, 5WMO, 6LHH, 6MNO, 6MKD, 6MKR, 7K80, 6PA1, 6RP9, 6RPA, 7LG0, 6VIU, 6UZN, 6VB6, 6VB5, 7MKB, 7N6E, 7N1A, 7N6D, 7P3D, 7N1F
Tfold failure*	6ILC, 6ILG, 6ILF, 6PBH, 6KVM
MHC-Fine failure*	6NF7, 6LF9, 6PBH, 6MP0, 7MJ8, 6LF8, 6E1I, 6ZKX

*Excluded only from the comparative benchmarking with the corresponding tool, due to runtime failure of that tool.

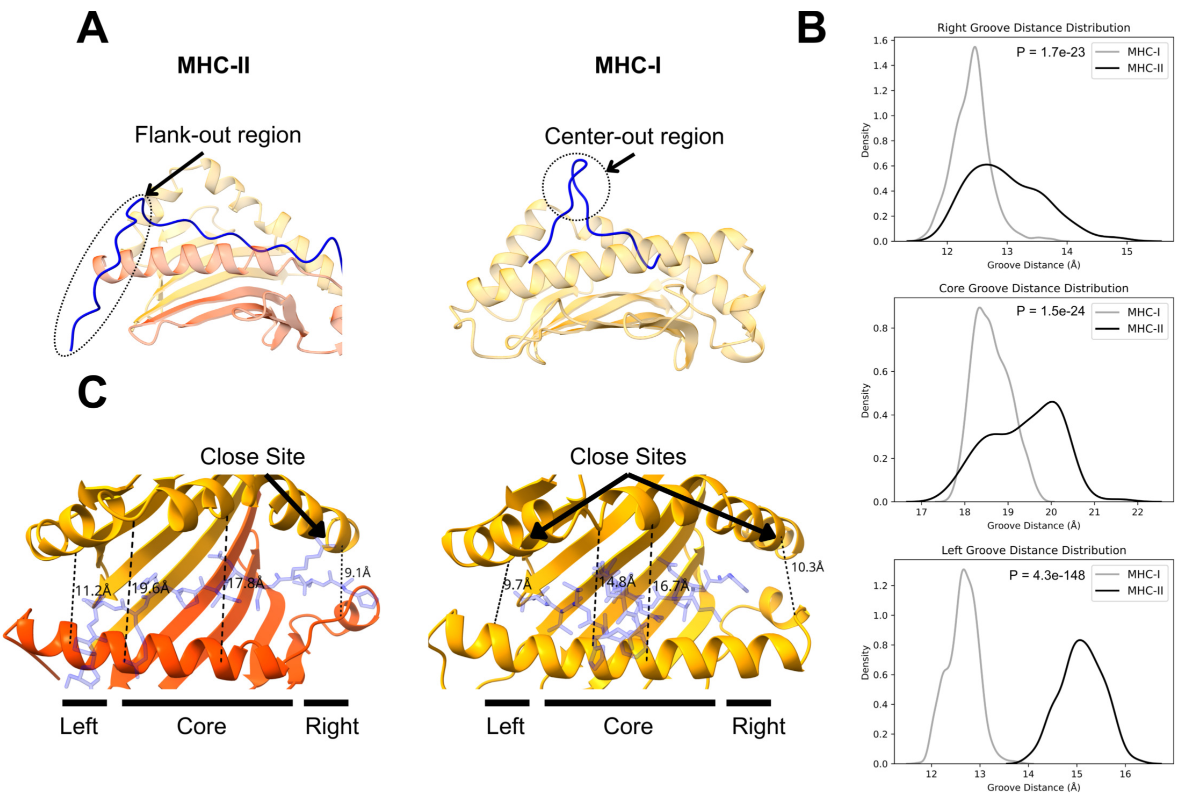

Figure S1: **Distinct binding properties of MHC-I and MHC-II.** (A) MHC-I tends to form a core-out conformation, whereas MHC-II adopts a flank-out conformation. (B) The binding groove of MHC-I has a smaller distance at both the right and left sites, while a larger central groove distance indicates the peptide's tendency to bulge outward. In contrast, MHC-II shows partial overlap with MHC-I in the right and core groove regions but exhibits complete separation in the left region due to its flank-out property. (C) Illustration of the binding groove regions in MHC structures: right (peptide entry site), core, and left (peptide exit site).

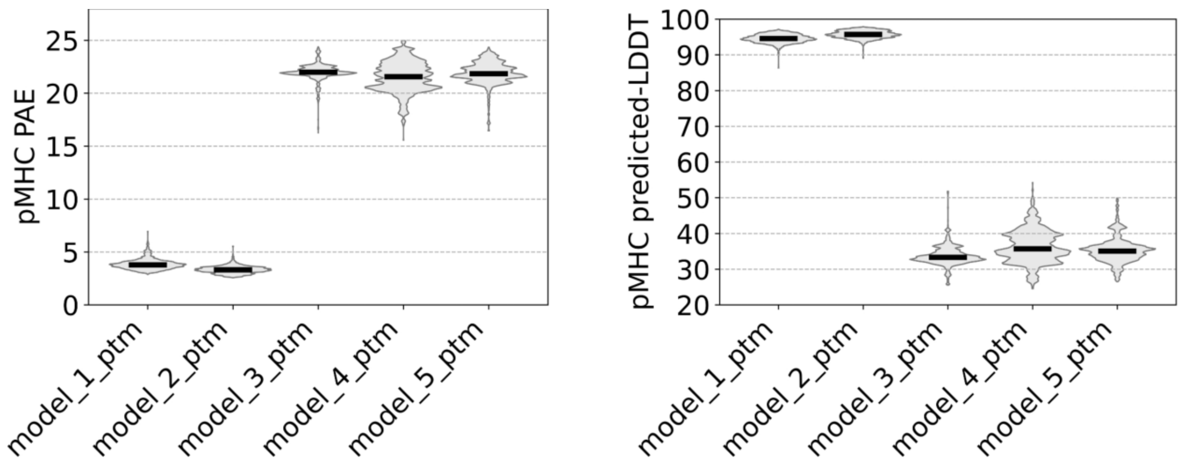

Figure S2: **AlphaFold2 performance.** The performance of different models in peptide-MHC structure prediction is visualized on the discovery set averaged over predictions on different numbers of templates (1 to 6).

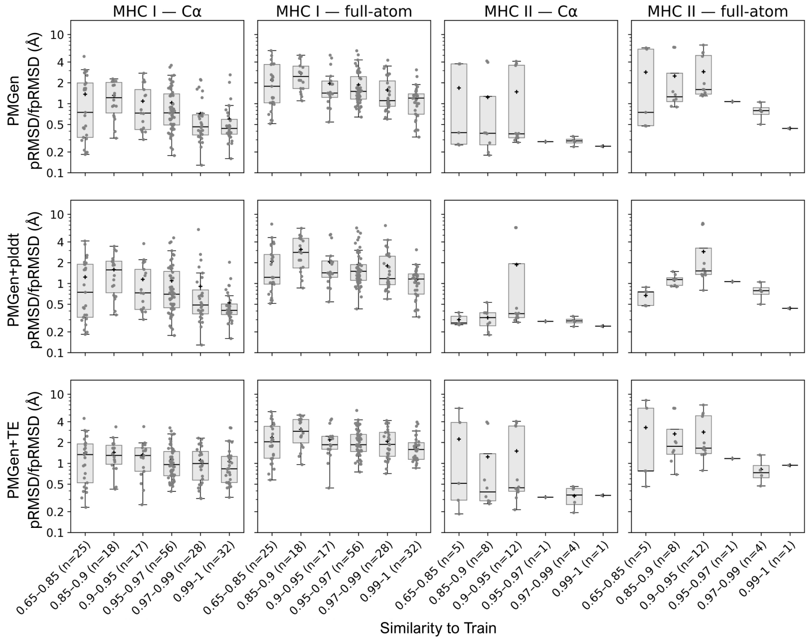

Figure S3: **PMGen performance across different levels of sequence similarity to the AlphaFold training set.**

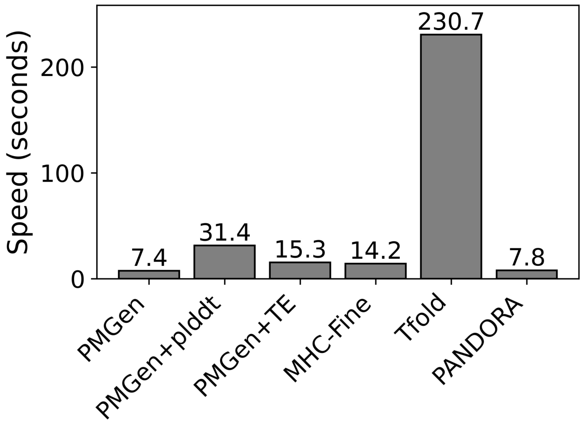

Figure S4: **Speed Benchmark**

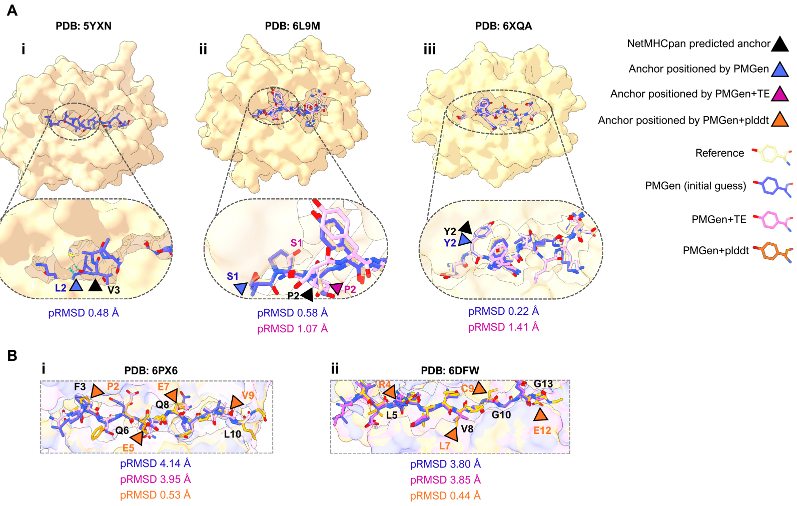

Figure S5: **Initial Guess mode anchor correction.** (A) Examples of PMGen structure predictions where: **i.** NetMHCpan predicted the wrong anchor, but PMGen (IG) positioned it correctly; **ii.** PMGen+TE agreed with the incorrectly predicted anchors from NetMHCpan, while PMGen (IG) positioned the anchor correctly; **iii.** PMGen+TE, PMGen, and NetMHCpan all correctly defined the anchor, but the core region in PMGen+TE was wrongly predicted due to the influence of engineered templates. (B) PMGen+pLDDT correctly identified the anchors using only pLDDT scores, whereas PMGen (IG) and PMGen+TE misplaced the anchors due to incorrect predictions from NetMHCpan. Cα-pRMSD values and amino acid identities are shown for PMGen (blue), PMGen+TE (pink), PMGen+pLDDT (orange), the reference structure (tan), and NetMHCpan (black).

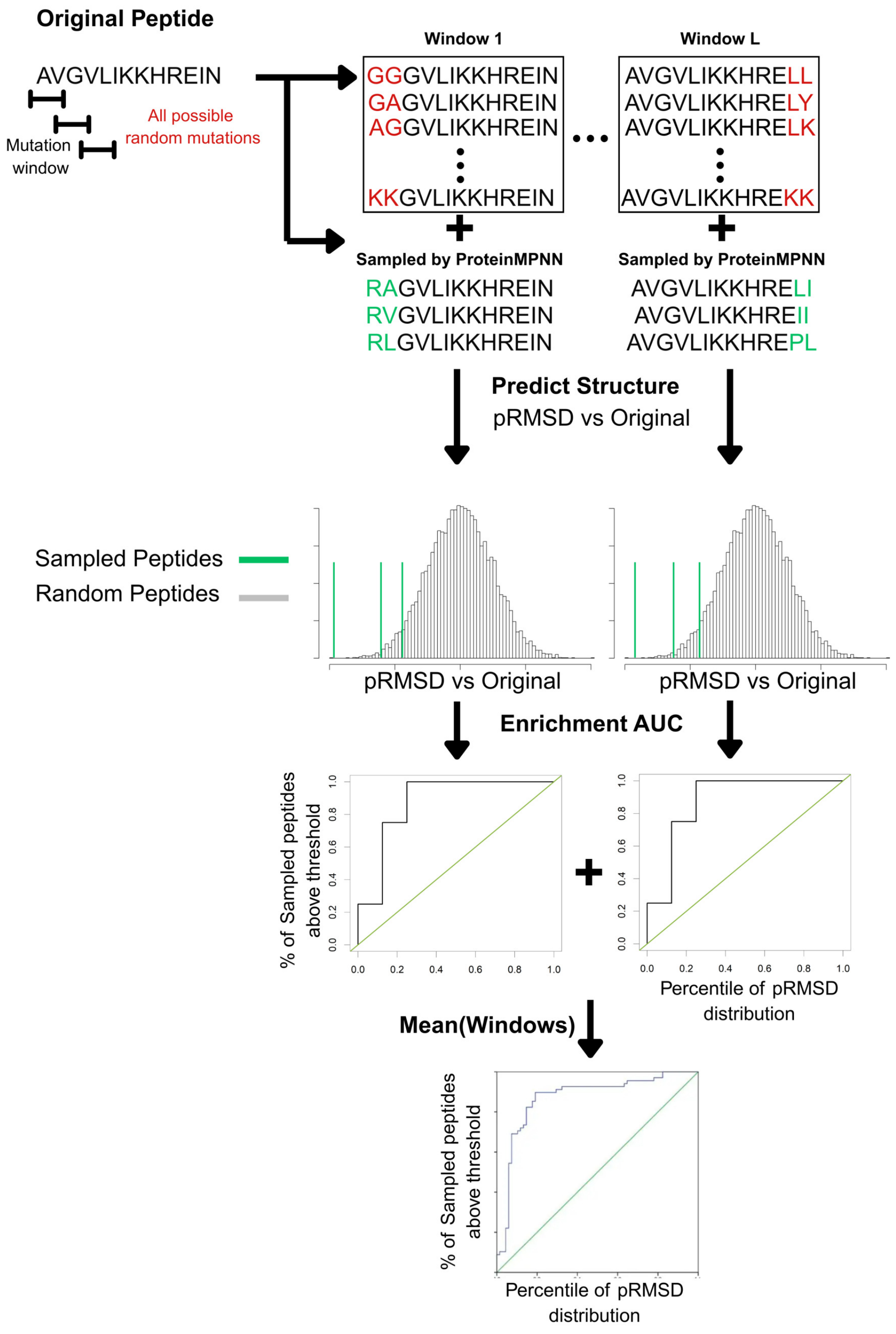

Figure S6: **Mutation screen analysis pipeline.**

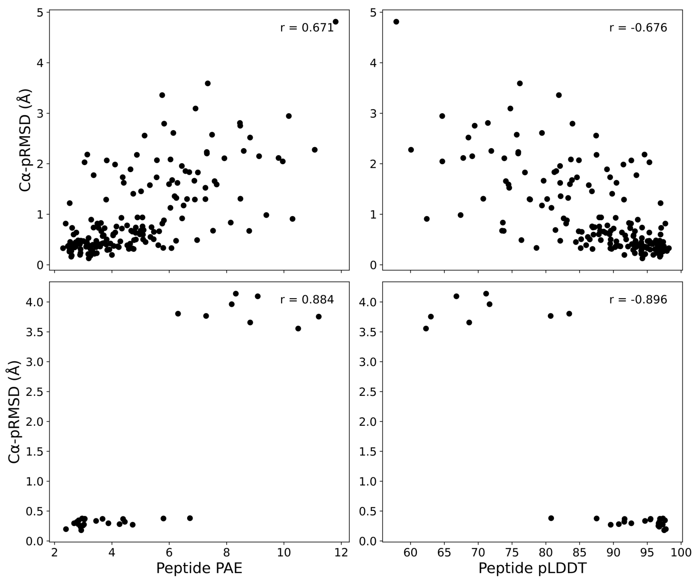

Figure S7: **PMGen Cα-pRMSD correlation with PAE and pLDDT.**

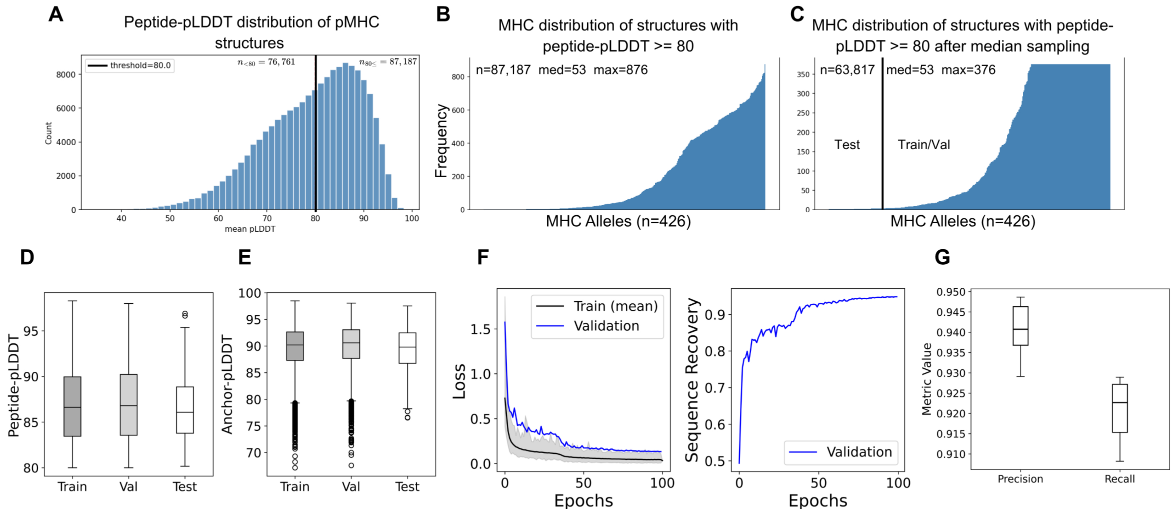

Figure S8: **ProteinMPNN fine-tuning with PMGen structures.** (A) Superset from which we select the dataset for fine-tuning of ProteinMPNN by a criterion of peptide $pLDDT \geq 80$ (vertical line). (B) Distribution of the count of generated structures from which those for fine-tuning are selected for each of the 426 alleles, sorted by structure count. (C) Distribution of the count of structures used for fine-tuning for each of the 426 alleles, but with the number of structures for dominant MHCs capped to a maximum of 376. (D-E) Distribution of peptide-pLDDT and anchor-pLDDT for train, validation and test datasets. (F) Training and validation loss (left) and validation sequence recovery (right) monitored over 100 epochs. (G) Precision and Recall on amino acid unmasking on test set peptides.

References

J. Dauparas, I. Anishchenko, N. Bennett, H. Bai, R. J. Ragotte, L. F. Milles, B. I. Wicky, A. Courbet, R. J. de Haas, N. Bethel, et al. Robust deep learning–based protein sequence design using proteinmpnn. *Science*, 378(6615): 49–56, 2022.

DTU Health Tech. Netmhciipan-4.3 server, 2025. Predicts peptide binding to HLA class II molecules using Artificial Neural Networks (ANNs).

E. Glukhov, D. Kalitin, D. Stepanenko, Y. Zhu, T. Nguyen, G. Jones, T. Patsahan, C. Simmerling, J. C. Mitchell, S. Vajda, et al. Mhc-fine: Fine-tuned alphafold for precise mhc-peptide complex prediction. *Biophysical Journal*, 2024.

D. F. Marzella, F. M. Parizi, D. v. Tilborg, N. Renaud, D. Sybrandi, R. Buzatu, D. T. Rademaker, P. A. 't Hoen, and L. C. Xue. Pandora: a fast, anchor-restrained modelling protocol for peptide: Mhc complexes. *Frontiers in Immunology*, 13:878762, 2022.

V. Mikhaylov, C. A. Brambley, G. L. Keller, A. G. Arbuiso, L. I. Weiss, B. M. Baker, and A. J. Levine. Accurate modeling of peptide-mhc structures with alphafold. *Structure*, 32(2): 228–241, 2024.

M. Mirdita, K. Schütze, Y. Moriwaki, L. Heo, S. Ovchinnikov, and M. Steinegger. Colabfold: making protein folding accessible to all. *Nature methods*, 19(6):679–682, 2022.

S. Mitternacht. Freesasa: An open source c library for solvent accessible surface area calculations. *F1000Research*, 5, 2016.

A. Motmaen, J. Dauparas, M. Baek, M. H. Abedi, D. Baker, and P. Bradley. Peptide-binding specificity prediction using fine-tuned protein structure prediction networks. *Proceedings of the National Academy of Sciences*, 120(9): e2216697120, 2023.

B. Reynisson, B. Alvarez, S. Paul, B. Peters, and M. Nielsen. Netmhcpan-4.1 and netmhciipan-4.0: improved predictions of mhc antigen presentation by concurrent motif deconvolution and integration of ms mhc eluted ligand data. *Nucleic acids research*, 48(W1): W449–W454, 2020.

F. Sievers, A. Wilm, D. Dineen, T. J. Gibson, K. Karplus, W. Li, R. Lopez, H. McWilliam, M. Remmert, J. Söding, et al. Fast, scalable generation of high-quality protein multiple sequence alignments using clustal omega. *Molecular systems biology*, 7(1):539, 2011.

M. Z. Tien, A. G. Meyer, D. K. Sydykova, S. J. Spielman, and C. O. Wilke. Maximum allowed solvent accessibilites of residues in proteins. *PloS one*, 8(11):e80635, 2013.

P. Wongklaew, S. Sriswasdi, and E. Chuangsuwanich. Mhcseqnet2—improved peptide-class i mhc binding prediction for alleles with low data. *Bioinformatics*, 40(1):btad780, 2024.

H. Xia, J. McMichael, M. Becker-Hapak, O. C. Onyeador, R. Buchli, E. McClain, P. Pence, S. Supabphol, M. M. Richters, A. Basu, et al. Computational prediction of mhc anchor locations guides neoantigen identification and prioritization. *Science immunology*, 8(82): eabg2200, 2023.
